# The Molecular and Neuropathological Consequences of Genetic Risk for Alzheimer's Dementia

**DOI:** 10.3389/fnins.2018.00699

**Published:** 2018-10-08

**Authors:** Shinya Tasaki, Chris Gaiteri, Sara Mostafavi, Philip L. De Jager, David A. Bennett

**Affiliations:** ^1^Rush Alzheimer's Disease Center, Rush University Medical Center, Chicago, IL, United States; ^2^Department of Neurological Sciences, Rush University Medical Center, Chicago, IL, United States; ^3^Department of Statistics, Medical Genetics, University of British Columbia, Vancouver, BC, Canada; ^4^Department of Neurology, Center for Translational and Computational Neuroimmunology, Columbia University Medical Center, New York, NY, United States; ^5^Cell Circuits Program, Broad Institute, Cambridge, MA, United States

**Keywords:** Alzheimer's disease, polygenic risk score, PheWAS, brain pathology, multi omics, epigenome, lifestyle, personality

## Abstract

Alzheimer's dementia commonly impacts the health of older adults and lacks any preventative therapy. While Alzheimer's dementia risk has a substantial genetic component, the specific molecular mechanisms and neuropathologies triggered by most of the known genetic variants are unclear. Resultantly, they have shown limited influence on drug development portfolios to date. To facilitate our understanding of the consequences of Alzheimer's dementia susceptibility variants, we examined their relationship to a wide range of clinical, molecular and neuropathological features. Because the effect size of individual variants is typically small, we utilized a polygenic (overall) risk approach to identify the global impact of Alzheimer's dementia susceptibility variants. Under this approach, each individual has a polygenic risk score (PRS) that we related to clinical, molecular and neuropathological phenotypes. Applying this approach to 1,272 individuals who came to autopsy from one of two longitudinal aging cohorts, we observed that an individual's PRS was associated with cognitive decline and brain pathologies including beta-amyloid, tau-tangles, hippocampal sclerosis, and TDP-43, *MIR132*, four proteins including VGF, IGFBP5, and STX1A, and many chromosomal regions decorated with acetylation on histone H3 lysine 9 (H3K9Ac). While excluding the *APOE/TOMM40* region (containing the single largest genetic risk factor for late-onset Alzheimer's dementia) in the calculation of the PRS resulted in a slightly weaker association with the molecular signatures, results remained significant. These PRS-associated brain pathologies and molecular signatures appear to mediate genetic risk, as they attenuated the association of the PRS with cognitive decline. Notably, the PRS induced changes in H3K9Ac throughout the genome, implicating it in large-scale chromatin changes. Thus, the PRS for Alzheimer's dementia (AD-PRS) showed effects on diverse clinical, molecular, and pathological systems, ranging from the epigenome to specific proteins. These convergent targets of a large number of genetic risk factors for Alzheimer's dementia will help define the experimental systems and models needed to test therapeutic targets, which are expected to be broadly effective in the aging population that carries diverse genetic risks for Alzheimer's dementia.

## Introduction

The ultimate goal behind large-scale sequencing studies in Alzheimer's dementia is to promote novel drug discovery efforts by identifying the origin of pathogenic mechanisms. However, despite gene discoveries from ever-larger sequencing studies, most clinical trials (Cummings et al., 2017) remain focused on the beta-amyloid pathway identified in familial Alzheimer's disease (AD) in the early 1990's (Goate et al., 1991; Strittmatter et al., 1993; Levy-Lahad et al., 1995; Sherrington et al., 1995). Several factors may be responsible for the limited translation of genetic findings to novel disease mechanisms. These include weak effects of most variants (Lambert et al., 2013), rare variants that have escaped detection (Sims et al., 2017), epigenomic changes associated with AD pathologies (De Jager et al., 2014), complexity of molecular systems supporting cognition (Gaiteri et al., 2016; Mostafavi et al., 2018), complexity of the AD phenotype (Farfel et al., 2016; Bennett, 2017), inadequacy of animal models based on these variants (Sabbagh et al., 2013; Burns et al., 2015; Foley et al., 2015), or external factors, such as economics of trial failures in Alzheimer's dementia (Doody et al., 2013, 2014; Cummings et al., 2014; Salloway et al., 2014).

Attempts to extract actionable molecular mechanisms from Alzheimer's dementia susceptibility variants have sought to unify genetic findings into coherent biological processes. Some variants appear to segregate into cell-specific functions (Raj et al., 2014), enriched in canonical (Karch and Goate, 2015) or data-driven pathway (Miller et al., 2013; Zhang et al., 2013). Other selected Alzheimer's dementia susceptibility variants show effects on brain structure (Sabuncu et al., 2012; Chauhan et al., 2015; Foley et al., 2017) or brain networks (Reiman et al., 2005; Filippini et al., 2009; Chhatwal et al., 2013; Zhang et al., 2015). At the same time, the biological significance of a portion of identified variants is unclear. This may be due to lack of associations with endophenotypes, which are specific cellular or molecular components of the disease that are more actionable targets for the activity triggered by variants. One direct way to connect variants to endophenotypes is to employ them as outcomes for a genome-wide association study (GWAS) (Flint et al., 2014). In practice, this approach is challenging as such phenotypes are generally not available for large cohorts. However, in a few reported cases, this approach led to the identification of several loci associated with endophenotypes. (Ramanan et al., 2015; Deming et al., 2017; Chibnik et al., 2018; Chung et al., 2018). These results generally provide additional candidate loci but do not clarify the effects of the GWAS hits for Alzheimer's dementia.

In this study, we attempted to discover actionable relationships between Alzheimer's dementia susceptibility variants and disease mechanisms by identifying the polygenic effects of genetic variants on clinical, molecular and neuropathological phenotypes. These endophenotypes are useful in defining convergent experimentally actionable targets for Alzheimer's dementia to aid the translation of current and future genetic findings into disease mechanisms that can be addressed therapeutically. We addressed the small effect sizes of recent genetic studies of Alzheimer's dementia by aggregating genetic variants into a total risk score calculated for each individual (polygenic risk score-PRS), and then searched for endophenotypes affected by this polygenic risk. Specifically, we compared predicted polygenic risk to a wide range of psychological, cognitive, behavioral, neuropathological, and molecular phenotypes that cover several aspects of cellular regulation, all measured in hundreds to more than a thousand individuals. PRS associations were found with epigenomes, a microRNA (miRNA), and proteins, all measured in the dorsolateral prefrontal cortex, as well as other neurodegenerative disease pathologies. These factors associated with polygenic risk showed differential genetic architecture to key components of Alzheimer's dementia such as tau-tangles, and together they may carry the genetic risk for Alzheimer's dementia to lead cognitive decline. Thus, we identified the molecular and pathological features which can provide novel targets for interventions or biomarkers backed with genetic evidence.

## Methods

### Cohort summaries for the religious orders study (ROS) and rush memory and aging project (MAP)

The ROS and MAP studies, based out of the Rush Alzheimer's Disease Center (RADC) in Chicago are two longitudinal, community-based aging studies collectively referred to as ROSMAP (Bennett et al., 2018), with many harmonized data measures. Together, these ongoing studies have enrolled >3,500 older persons without dementia, all of whom have agreed to brain donation and annual detailed clinical evaluation, cognitive testing and blood donation. As of March 2018, a total of 2,093 individuals were genotyped. We used data from 1272 deceased individuals with genotype measurement in this study. The brain autopsies were approved by a board-certified neuropathologist. Of these,1,260 are non-Latino White, 11 are Latino, and 1 is African American. Of these, 847 individuals were female and 425 were male (Supplementary Table 1). All omics analyses were performed on the dorsolateral prefrontal cortex (DLPFC). Cognitive decline and pathological indices utilized in comparison to polygenic risk come directly from measurements provided by this cohort. All phenotypes and omics data are shared widely with a data use agreement through the RADC Resource Sharing Hub (www.radc.rush.edu).

### Standard protocol approvals, registrations, and patient consents

The parent cohort studies and sub-studies were approved by Rush University Medical Center Institutional Review Boards. Participants provided written informed consent and all participants signed an Anatomic Gift Act for brain donation.

### Clinical evaluation

For each participant, a comprehensive clinical evaluation was administered at baseline and during each annual follow-up visit. Details of the evaluations were described previously (Wilson et al., 2002, 2015b; Bennett et al., 2006). Briefly, the cognitive battery contains a total of 21 cognitive performance tests, of which 19 are in common between ROS and MAP. Of these 17 are used to construct a global composite measure of cognitive function (Wilson et al., 2015a) and assess 5 dissociable cognitive domains including, episodic memory (7 measures), semantic memory (3 measures), working memory (3 measures), perceptual speed (2 measures), and visuospatial ability (2 measures) (Wilson et al., 2015b). To minimize the floor and ceiling effects, composite measures were used to examine the longitudinal cognitive decline. The longitudinal rate of decline was computed for each participant using linear mixed models with adjustment for the effects of age, sex, and education, which estimate person-specific residual slopes (Wilson et al., 2007).

The evaluation also provides clinical diagnoses of Alzheimer's dementia, and other causes of dementia, major depression, and stroke, as well as extensive characterization of lifestyle, personality, and other medical conditions, as described (Bennett et al., 2012a,b). The complete list of clinical variables is in Supplementary Table 2.

### TAU and beta-amyloid measurement

Tissue was dissected from 8 brain regions to quantify the load of parenchymal deposition of beta-amyloid by image analysis and the density of abnormally phosphorylated paired helical filament tau (PHFtau)-positive neurofibrillary tangles by stereology. Tissue sections (20 μm) were stained with antibodies against beta-amyloid protein and PHFtau protein, and quantified using image analysis and stereology, as previously described (Bennett et al., 2006, 2012c; Schneider et al., 2012; Boyle et al., 2013). Pathologic AD was generated from five regions which were stained with modified Bielschowski. Other pathologic diagnoses were made as described (Boyle et al., 2018). The complete list of brain pathologies assessed in this study is in Supplementary Table 2.

### Genotype processing in ROS and MAP

Genotyping of the ROS and MAP subjects was performed on the Affymetrix Genome-Wide HumanSNP Array6.0 (*n* = 1709) or the Illumina OmniQuad Express platform (*n* = 384). Genotypes were measured using DNA extracted from peripheral blood mononuclear cells or frozen brain tissue, and quality control steps were performed as described previously (Shulman et al., 2013). Dosages for all single nucleotide polymorphisms (SNPs) on the 1000 Genomes reference were imputed using BEAGLE (Browning and Browning, 2009) (version 3.3.2; 1000 Genomes Project Consortium interim phase I haplotypes, 2011 Phase 1b data freeze). The coordinates of SNPs were updated with dbSNP Build 150. SNPs with minor allele frequency >0.05 and info score >0.3 were used for the subsequent analyses, resulting in 7,159,943 SNPs.

### Methylation processing

Details on DNA methylation data were published previously (De Jager et al., 2014). Briefly, DNA from 740 individuals was extracted from DLPFC using the Qiagen QIAamp DNA mini protocol. DNA methylation data were generated using Illumina Infinium HumanMethylation450 BeadChip. The beta methylation values were adjusted using linear regression with the following variables: sex, age at death, cell epigenotype specific indexes, the first three genotyping principal components, post-mortem interval (PMI), array positions, study index and batch. After normalization, we performed an initial data reduction using the minfi Bioconductor package (Aryee et al., 2014) to collapse adjacent probes with similar methylation levels into single units as described previously (Gaiteri et al., 2018). This resulted in ~130,000 methylation loci.

### Histone acetylation processing

Details on histone acetylation data were previously published (Klein et al., 2018; Tasaki et al., 2018). Briefly, Chromatin Immunoprecipitation (ChIP) assay using anti-H3K9Ac mAb coupled with sequencing was performed in gray matter from 669 biopsies of DLPFC. The resulting datasets included 26,384 peaks. Read counts from 641 individuals which had genotype data were transformed by adding a constant (0.5) and then log2 transformed with adjustment of the effective library sizes [as estimated by trimmed mean of *M*-values (TMM) scale-normalization using edgeR software; Robinson et al., 2010]. The read counts were then quantile normalized. To remove outlier samples, we followed the procedure used by the Genotype-Tissue Expression project (GTEx Consortium et al., 2017). Specifically, the statistic di was calculated as the correlation between each sample and the median of all samples. We excluded samples with a di value outside of 1.5x of the lower interquartile range. Nine samples were removed. We adjusted the data for the following variables using linear regression: sex, age at death, the first three genotyping principal components (PCs), PMI, study index (ROS or MAP) and the data quality metrics which we found to be strongly correlated with the first PC (mean fold enrichment, total number of reads, 50% quantile of the mapping quality of all uniquely mapped reads, non-redundant fraction and PCR batch).

### RNAseq processing

Details on RNAseq data were previously published (Ng et al., 2017; Mostafavi et al., 2018). Briefly, RNA was extracted from DLPFC region of 540 individuals. The reads were aligned to the reference genome using Bowtie (Langmead et al., 2009) and the expression FPKM (fragment per kilobase of million) values were estimated using RSEM (Li et al., 2011). Samples from 494 individuals which had genotype data and passed the expression outlier test were further normalized. We kept only highly expressed genes (mean expression >2 FPKM), which resulted in 13,484 genes. The FPKM values were log transformed and the data were adjusted using linear regression for the following variables: sex, age at death, first three genotyping PCs, PMI, RIN (RNA integrity number), study index (ROS or MAP) and batch.

### Identifying molecular systems

Comparisons between every DNA methylation locus (~130,000), histone acetylation peak (~26,000), and expressed genes (~13,000) with the polygenic risk score for Alzheimer's dementia (AD-PRS) presents a large multiple testing burden. Therefore, we followed the standard practice of reducing DNA methylation, histone acetylation, and gene expression to comethylated, coacetylated, or coexpressed systems, each of which was composed of variables with similar patterns of methylation, acetylation, or expression, measured across all individuals. These systems are sometimes referred to as modules (Zhang and Horvath, 2005). In order to statistically identify groups of comethylated, coacetylated, or coexpressed genes we use the consensus clustering approach SpeakEasy (Gaiteri et al., 2015). This method was used because: (1) unlike hierarchical clustering approaches, SpeakEasy does not require any manual parameter tuning or threshold selection; (2) it has demonstrated the highest recorded performance on synthetic clustering benchmarks; (3) it provides accurate recovery of biological gold standards; and (4) it identifies clusters which are less likely to be influenced by statistical or data artifacts due to its stochastic nature and consensus clustering. This method operates on the DNA methylation locus-DNA methylation locus, histone acetylation peak-histone acetylation peak, or gene expression-gene expression Pearson correlation matrix to identify clusters or modules of coacetylated/comethylated/coexpressed variables. Using SpeakEasy we identified 58 DNA comethylation modules, 80 histone coacetylation modules, and 49 coexpression modules (Supplementary Table 3). The modules encompass a wide range of functional cellular systems, which in many cases correspond to canonical pathways, but also provide measures of robust cellular systems that are less well annotated (Gaiteri et al., 2014). We computed the normalized acetylation/methylation/expression level by subtracting the mean level for that variable across all individuals and dividing it by the standard deviation. Then, we summarized the composite metric of each module in each individual by computing the mean of the normalized levels across all variables in that module.

### miRNA-array

The miRNA expression profiles were collected for about 700 miRNAs from 734 frozen post-mortem DLPFC samples using the NanoString nCounter miRNA expression assay (Patrick et al., 2017). We pre-processed the dataset to retain all miRNAs that had a call rate of 95% and an absolute value above 15 in at least 50% of the samples. The batch effects (cartridges) were corrected using Combat (Johnson et al., 2007). The data were adjusted for the following variables: sex, age at death, PMI, RIN, years of education and the first three genotyping PCs. The pre-processing resulted in 292 miRNAs encoded in human genome in 655 subjects.

### Targeted liquid chromatography (LC) selected reaction monitoring (SRM) proteomics

SRM proteomics was performed using frozen DLPFC tissue for 67 proteins selected by the consortium members of Accelerating Medicines Partnership for Alzheimer's Disease (AMP-AD; https://www.synapse.org/#!Synapse:syn2580853) (Yu et al., 2018). The samples were prepared for LC-SRM analysis using the standard protocol described previously (Petyuk et al., 2010; Andreev et al., 2012). Briefly, the abundance of endogenous peptides was quantified as a ratio to spiked-in synthetic peptides containing stable heavy isotopes. The “light/heavy” ratios were log2 transformed and shifted such that median log2-ratio was zero. Data were adjusted for the following variables: sex, age at death, PMI, experimental batch, years of education and the first three genotyping PCs. The pre-processing resulted in 67 proteins in 765 subjects.

### Polygenic risk score generation

The genetic variants comprising our AD-PRS were identified based on GWAS data from the International Genomics of Alzheimer's Project (IGAP) (Lambert et al., 2013). IGAP is the largest aggregation collection of genomic data for Alzheimer's dementia. The IGAP study conducted the two-stage meta-analysis with a total of 25,580 Alzheimer's dementia cases and 48,466 controls. The summary statistics from the entire cohort are available for 11,632 SNPs that indicate *p* < 0.001 in the first stage of the IGAP study. To select SNPs for AD-PRS, we used the entire cohort summary statistics of 6,411 SNPs that showed *p* < 0.001 in the entire cohort. Since SNPs located in the Apolipoprotein E (*ApoE*) or translocase of outer mitochondrial membrane 40 (*TOMM40*) were only measured in the first stage (Lambert et al., 2013), the first stage summary statistics of three SNPs (rs769449, rs769450, and rs429358) in the region were also added to the initial set of SNPs. SNPs in linkage disequilibrium (LD) were pruned with the threshold of *R*^2^ > 0.1 and the window of 2,000 kb using LD estimates based on all 2,093 genotyped ROSMAP participants. This resulted in 457 independent SNPs which included rs769449 and rs769450 for *ApoE*/*TOMM40* region (Supplementary Table 4). We calculated AD-PRS for 1,272 deceased individuals as an average of the number of risk-increasing allele weighted by the summary statistic using PRSice-2 software (Euesden et al., 2015). An AD-PRS without two SNPs located in *ApoE*/*TOMM40* region and an AD-PRS consisting of two *ApoE*/*TOMM40* SNPs were also generated in the same procedure as above. We then scaled AD-PRSs by subtracting the mean AD-PRSs across all individuals and dividing by the standard deviation.

### Statistical analysis

Linear or logistic regression models were used for testing associations between AD-PRS and continuous or categorical traits, respectively. The following variables were removed from the continuous traits using linear regression leaving the residuals for use in the association test with AD-PRS: age at measurement, sex, years of education and the first three genotyping PCs. For categorical traits, these covariates were included in the logistic regression model.

### Estimation of AD-PRS effect explained by endophenotypes

To evaluate the proportion of AD-PRS effect on global cognitive decline explained by endophenotypes, we compared the variance of global cognitive decline explained by AD-PRS and that given each molecular phenotype as follows:

First, the total variance of global cognitive decline was computed as the total sum of squares (SS),

$$\begin{array}{l} SS_{total}=\underset{i}{\sum }{\left(y_{i}-\bar{y}\right)}^{2} \end{array}$$

where *y*_*i*_ is global cognitive decline for *i* individual, and $\bar{y}$ is an average of global cognitive decline. Then global cognitive decline was regressed with AD-PRS alone,

$$\begin{array}{l} f^{1}\left(ADPRS\right)=a_{1}+b_{1}*ADPRS+\varepsilon_{1} \end{array}$$

where *a*_1_ is an intercept, *b*_1_ is a coefficient for AD-PRS, and ε_1_ is an error term. The SS for the residual of the first model was computed as

$$\begin{array}{l} SS_{residual}^{1}=\underset{i}{\sum }{\left(y_{i}-f_{i}^{1}\right)}^{2} \end{array}$$

and then the proportion of variance explained by AD-PRS was calculated as

$$\begin{array}{l} PVE^{1}=1-\frac{SS_{residual}^{1}}{SS_{total}}. \end{array}$$

Next, global cognitive decline was regressed with AD-PRS and a mediator (M),

$$\begin{array}{l} f^{2}\left(ADPRS,M\right)=a_{1}+b_{2}*ADPRS+c_{2}*M+\varepsilon_{2} \end{array}$$

where *a*_2_ is an intercept, *b*_2_ is a coefficient for AD-PRS, *c*_2_ is a coefficient for a mediator, and ε_2_ is an error term. The SS for the residual of the second model was computed as

$$\begin{array}{l} SS_{residual}^{2}=\underset{i}{\sum }{\left(y_{i}-f_{i}^{2}\right)}^{2} \end{array}$$

and then the proportion of variance explained by AD-PRS was calculated as

$$\begin{array}{l} PVE_{ADPRS}^{2}=\left(1-\frac{SS_{residual}^{2}}{SS_{total}}\right)*RI_{ADPRS} \end{array}$$

The component *RI*_*ADPRS*_ is the relative contribution of AD-PRS to the variance explained by the second model and was calculated using the variance decomposition method proposed by Chevan and Sutherland (1991). The method is implemented in relaimpo R package (Grömping, 2006). Lastly, the percent of AD-PRS effect explained by a mediator (PAEM) was computed as

$$\begin{array}{l} PAEM=\frac{PVE^{1}-PVE_{ADPRS}^{2}}{PVE^{1}}*100. \end{array}$$

To assess whether PAEM is greater than random, the above procedure was performed with permutated mediator values 10,000 times in order to estimate a null distribution.

### Conditional independence testing

A conditional independence test was carried out to examine the null hypothesis stated as: “global cognitive decline is independent of AD-PRS given a conditioning set of endophenotypes.” The *p*-value was calculated using an *F*-test by comparing a linear regression model based on the conditioning set of endophenotypes against a model where the regressors are both AD-PRS and the conditioning set. The R MXM package was used for this analysis (Lagani et al., 2017).

### Building a trait MAP using T-distributed stochastic neighbor embedding (t-SNE)

We generated a trait map by calculating the distance between traits in their associations with SNPs in the AD-PRS to understand which set of traits is associated with the same set of SNPs in the AD-PRS. To calculate the location of each trait in genotype space, we first determined their associations with SNPs in the AD-PRS. We calculated pairwise correlation of each SNP and each of the traits associated with AD-PRS (without including the SNPs of interest in the AD-PRS). If the AD-PRS-associated trait was negatively correlated with AD diagnosis, the sign of association statistics was reversed to align SNP effect across traits. After binarizing the association statistic of each SNP according to its direction of effect, the distance between traits was calculated using the Jaccard index. To project the distance between traits on to two-dimensional, t-SNE (van der Maaten and Hinton, 2008) was performed with perplexity of 5 and 10,000 iterations.

### Identification of SNP groups

To investigate subgroups of SNPs in the AD-PRS that showed distinct association patterns with traits, we clustered SNPs used in the AD-PRS based on the associations to traits. Using the same approach as for building the trait map above, we calculated the distance between SNPs using Jaccard index given binarized association statistics. Based on the distance matrix, four SNP clusters were identified using the SpeakEasy consensus clustering method (Gaiteri et al., 2015).

### Gene ontology (GO) enrichment analysis for SNP groups

To examine whether SNP groups were involved in particular biological processes, we performed SNP-based GO enrichment analysis. GO gene sets were downloaded from MSigDB v6.1 (Subramanian et al., 2005; Liberzon et al., 2015). The GREAT algorithm (McLean et al., 2010) was used for the enrichment analysis of *cis* regions of SNP groups with gene sets. The BSgenome.Hsapiens.UCSC.hg19 and TxDb.Hsapiens.UCSC.hg19.knownGene R packages were used for background information. The genomic region for each GO was defined as follows. The genomic region from 1,000 kb upstream of transcriptional start site (TSS) to 1,000 kb downstream of transcriptional end site (TES) was assigned for each gene in GO. If other genes were present within 1,000 kb upstream of TSS or 1,000 kb downstream of TES of the gene of interest, the genomic region assigned for the gene was truncated at the point where the coding regions of other genes start. The genomic regions for all genes in each GO were then merged. Finally, the enrichment of SNPs in the genomic region assigned to each GO was evaluated by a binomial test. The Enrichment Map was utilized for visualization of the results (Merico et al., 2010).

### Partitioning heritability analysis

We conducted partitioning heritability analysis via the LD score regression (Finucane et al., 2015) to examine whether genetic risk variants for Alzheimer's dementia are enriched in the *cis-*regions of histone coacetylation modules. We tested for: (1) enrichment of Alzheimer's dementia heritability in the genomic regions with AD-PRS-associated histone coacetylation modules; (2) enrichment of Alzheimer's dementia heritability in the *cis-*histone acetylation quantitative trait loci (hQTL) for the histone acetylation peaks belonging to the AD-PRS-associated histone coacetylation modules. The list of hQTLs was obtained from the Brain xQTL Serve (Ng et al., 2017) and hQTLs exceeding Bonferroni-corrected threshold were used. LDSC v1.0.0 was downloaded from https://github.com/bulik/ldsc and the partitioning heritability analysis was carried out with its default parameter setting.

### Genomic annotation enrichment

Next, we tested whether histone coacetylated modules associated with the AD-PRS were localized to specific genomic regions. We conducted the enrichment analysis of histone coacetylated peaks with 15 chromatin states or seven histone marks from the mid-frontal gyrus (Roadmap Epigenomics Consortium et al., 2015). We employed Fisher's exact test as implemented by R LOLA package (Sheffield and Bock, 2016). The genomic regions of all 26,384 H3K9Ac peaks detected in ROSMAP data were used as a background region set.

### Identification of histone peaks associated with gene expression

We calculated Pearson's correlation between gene expression levels and histone acetylation peaks using the MatrixEQTL software (Shabalin, 2012) to identify functional histone peaks as described previously (Tasaki et al., 2018). We focused on histone acetylation peaks which were located within 1 Mbp upstream or downstream regions of the TSSs. This correlation analysis resulted in 479,003 tests in total. To handle outliers conservatively, mRNA levels and quantities of epigenomic peaks were quantile-normalized before the cross-omics mapping. We set significance criteria at *p* < 1.0 × 10^−7^ based on Bonferroni correction, which resulted in 1,893 peaks associated with gene expression levels (eQTH). The enrichment of eQTHs in each module was evaluated using the hypergeometric test.

### Gene ontology analysis for the genes associated with eQTHs

We performed GO enrichment analysis of the genes associated with eQTHs in the AD-PRS-associated histone modules to understand the biological processes possibly regulated by the modules. GO gene sets were downloaded from MSigDB v6.1 (Subramanian et al., 2005; Liberzon et al., 2015). The enrichment of GO terms was evaluated using the hypergeometric test with 12,609 unique Entrez genes detected in the RNAseq data as a background.

## Results

### Molecular and neuropathologic phenotypes associated with a polygenic risk score

We identified endophenotypes associated with the AD-PRS to aid in understanding the biological processes affected by genetic risk for Alzheimer's dementia. We examined a wide range of possible effects in several categories of endophenotypes, which included 3 clinical diagnoses, 6 cognitive decline measures, 8 lifestyle and personality traits, 14 medical conditions, 13 brain pathologies, and 5 types of omics measurements from the DLPFC. Details of these endophenotypes, such as their sub-components and methods of acquisition are described in the Methods section and in the Supplementary Table 2. In total, with pathological, cognitive and behavioral factors, 590 variables were tested for the association with the AD-PRS. In our tests, we accounted for sex, age, education, and the first three principal components of genotype. We adopted *p*-value of 0.05/590 as a Bonferroni-corrected significance threshold (*p* < 8.5 × 10^−5^).

**Figure 1:**
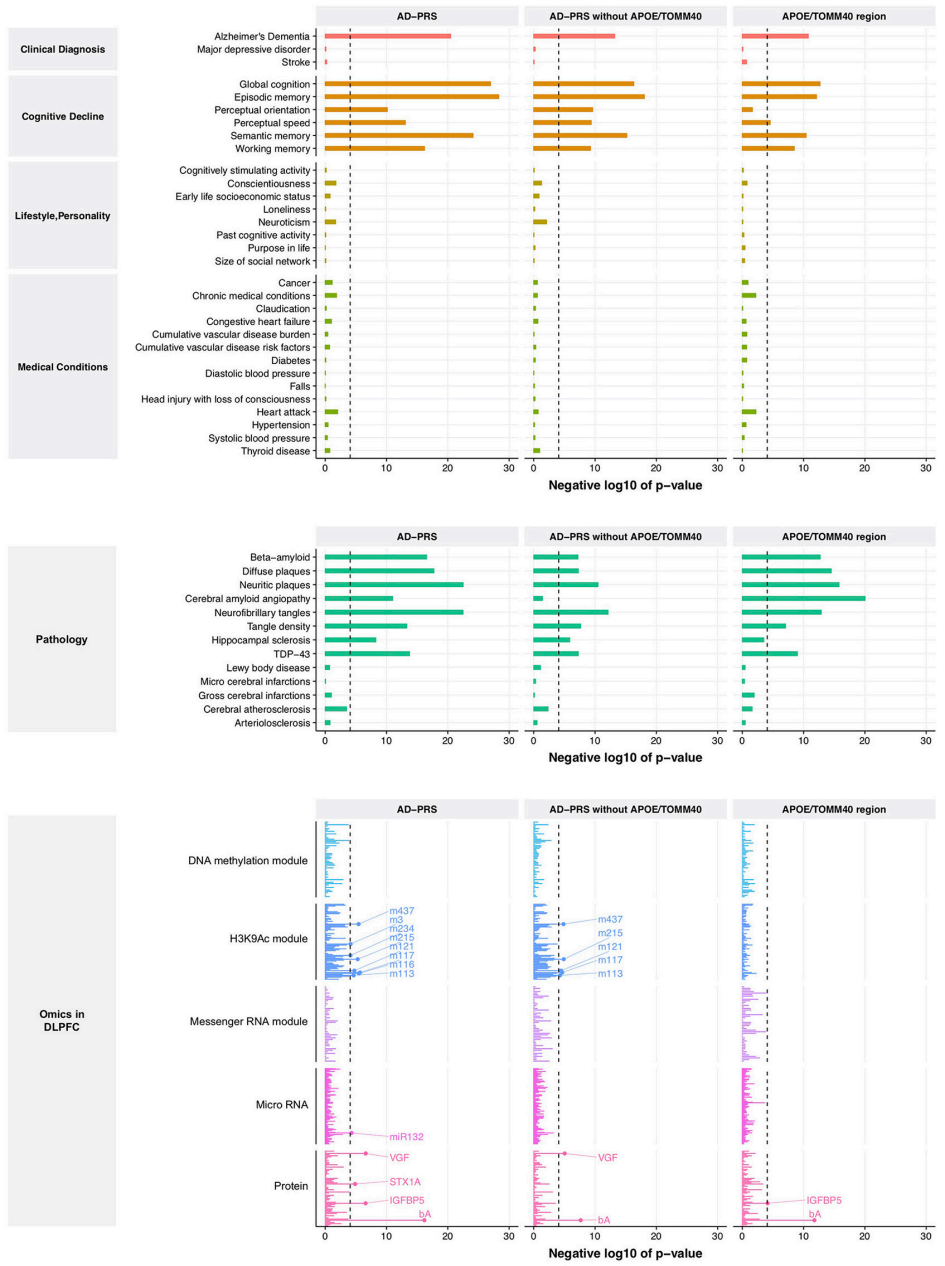
Association between AD-PRS and clinical phenotypes, neuropathologies, and molecular signatures. A dash line represents Bonferroni-corrected *p*-value at 0.05.

From this comparison to genetic risk, we identified 1 clinical diagnosis, 6 cognitive decline measures, 8 brain pathologies, 4 proteins, 1 miRNA (*MIR132*), and 8 histone coacetylation modules (*p* < 8.5 × 10^−5^) (Figure 1, left lane and Supplementary Table 5). Interestingly, no variables from lifestyle, personality, medical condition, DNA comethylation modules, and mRNA coexpression modules exceeded the significance criteria despite the fact that we have previously reported associations of lifestyle and personality factors with Alzheimer's dementia, and methylation and mRNA coexpression modules association with cognitive decline and AD pathology. Since the *APOE*/*TOMM40* region contains the strongest genetic risk factor for Alzheimer's dementia, we examined the effect of *APOE*/*TOMM40* for these associations by generating an AD-PRS without *APOE*/*TOMM40* SNPs and as well as examining the independent contribution of *APOE*/*TOMM40* SNPs. The AD-PRS without *APOE*/*TOMM40* SNPs was still associated with AD diagnosis, cognitive decline, proteins and histone coacetylation modules, and brain pathologies - except for cerebral amyloid angiopathy (Figure 1, middle lane). Interestingly, hippocampal sclerosis, VGF (non-acronymic, also called “VGF nerve growth factor inducible protein”), and histone coacetylation modules were only associated with the AD-PRS without *APOE*/*TOMM40* SNPs. Conversely, cerebral amyloid angiopathy and insulin like growth factor binding protein 5 (IGFBP5) were only associated with *APOE*/*TOMM40* SNPs (Figure 1, right lane), suggesting that the genetic effects of 457 SNPs on these variables are contingent on *APOE*/*TOMM40*. To investigate this possibility and further increase the robustness of our results, the polygenic nature of these associations was examined by varying *p*-value threshold for SNP inclusion in the AD-PRS, which altered the number of SNPs included in the model. The association with cerebral amyloid angiopathy was attenuated by increasing the number of SNPs in the AD-PRS (Supplementary Figure 1), indicating that it is subjected to monogenic influence from *APOE*/*TOMM40* SNPs. The other associations became stronger as the number of SNPs included in the AD-PRS increased (Supplementary Figure 1), which indicated polygenic influences from Alzheimer's dementia-associated SNPs as suggested previously (Mormino et al., 2016; Desikan et al., 2017).

### Identification of the endophenotypes that convey polygenic risk for cognitive decline

The strongest AD-PRS effect among the clinical phenotypes we tested in Figure 1 was on cognitive scores. Specifically, the individuals with a high AD-PRS showed a rapid decline in global cognitive scores, explaining 9.5% of variance after first accounting for sex, age, education, and the first three genotyping PCs (Supplementary Figure 2A). Interestingly, the association of AD-PRS with global cognitive decline remained significant after accounting for the clinical diagnosis of AD (Supplementary Figure 2B), which is the primary phenotype used in the IGAP study. This suggested that the AD-PRS captures a genetic architecture that has an effect not only on clinical AD but also other processes that contribute to cognitive decline.

**Figure 2:**
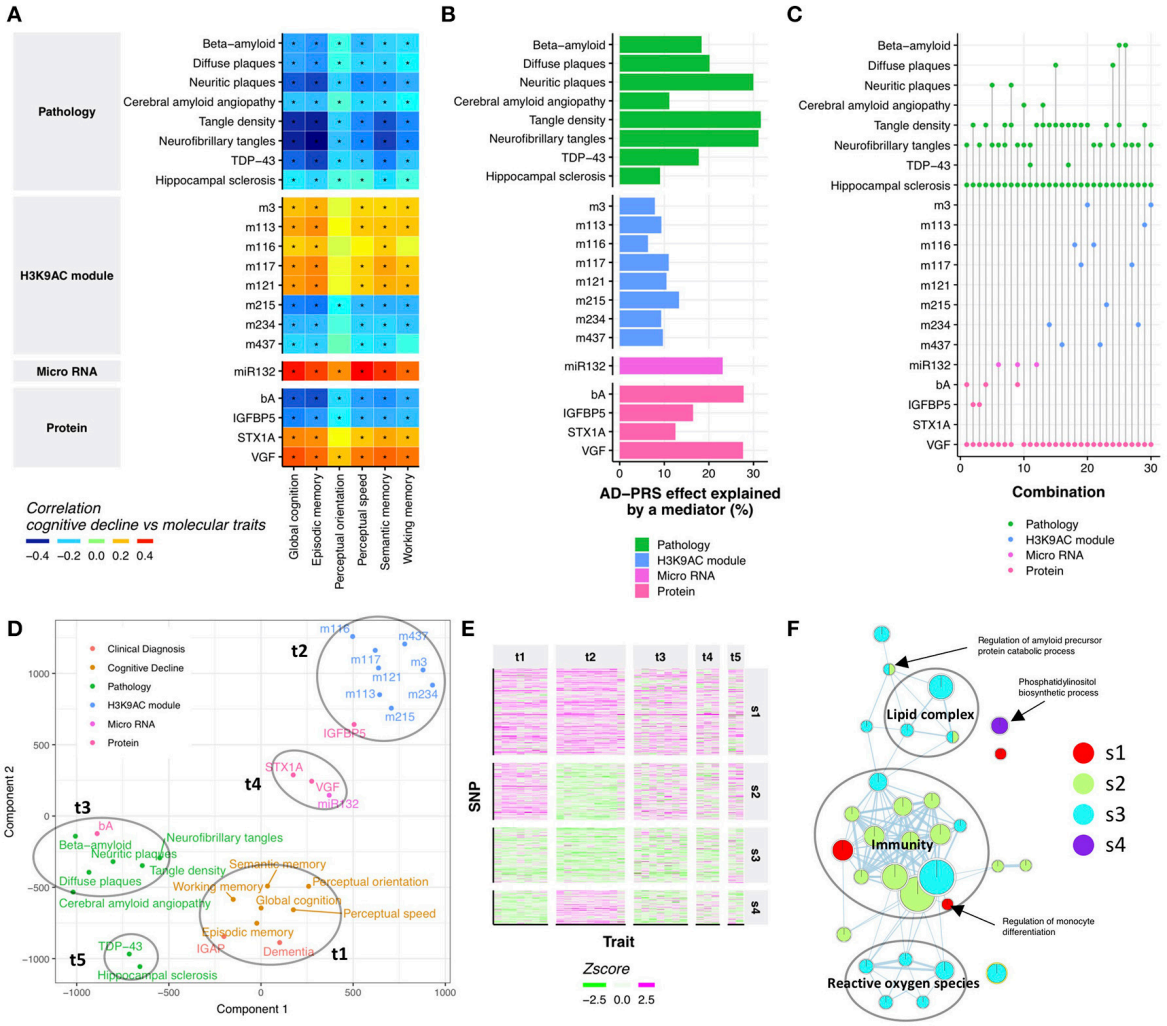
Neuropathological and molecular traits mediating AD-PRS **(A)**. Correlation between cognitive decline and AD-PRS-associated neuropathologies and molecular signatures. An asterisk indicates Bonferroni-corrected *p* < 0.05 **(B)**. The effect of AD-PRS on global cognitive decline explained by an endophenotype **(C)**. The sets of endophenotypes explain the effect of AD-PRS on global cognitive decline. The endophenotypes connected with a vertical line indicates the set of variables that made relationship between AD-PRS and global cognitive decline independent (*p* > 0.05) **(D)**. Traits map based on genetic associations. The trait map is generated by calculating the distance between traits in terms of genetic associations. The distance between traits was calculated using the Jaccard index and projected on to two-dimensional by t-SNE **(E)**. Heatmap of trait-SNP associations. The SNPs in the AD-PRS were clustered using the SpeakEasy consensus clustering method **(F)**. GO enrichment map for SNP clusters. GO enrichment for SNP clusters were conducted using the GREAT algorithm and the significant associations (FDR < 0.05) were visualized by EnrichmentMap.

Cognitive decline is the major source of disability in Alzheimer's dementia, yet its molecular origin is largely unknown (Boyle et al., 2013). Therefore, we examined which endophenotypes in ROSMAP may account for the relationship between the AD-PRS and cognitive decline. First, we performed a correlation analysis between AD-PRS-associated endophenotypes and cognitive decline to identify endophenotypes which might be potentially responsible for the relationship of AD-PRS to cognitive decline. All tested endophenotypes were associated with at least one cognitive decline measure (Figure 2A), which suggested that the effect of AD-PRS on cognitive decline could be explained by these endophenotypes. To gauge the magnitude of this effect, we contrasted the variance of global cognitive decline explained by the AD-PRS before and after controlling for each endophenotype. AD-PRS-associated brain pathologies and molecular hallmarks significantly attenuated the effects of AD-PRS on global cognitive decline (permutation *p*-value after Bonferroni correction <0.05; nominal *p* < 0.0024; Figure 2B), which indicated that the endophenotypes could be involved in the translation of genetic risk to cognitive decline. We observed stronger effects in beta-amyloid, tau-tangles and VGF, which explained approximately 30% of AD-PRS effect on global cognitive decline (Figure 2B).

Although the effects of attenuation were greater than expected at random, no endophenotype could completely diminish the associations between AD-PRS and cognitive decline (Supplementary Figure 3). This suggests that AD-PRS could be mediated by multiple molecular pathways. Therefore, we hypothesized that a combination of endophenotypes could explain the association between AD-PRS and global cognitive decline. We tested this hypothesis using a conditional independence test based on 404 samples where all endophenotypes were available. We found that at least four variables were necessary to make the relationship between AD-PRS and global cognitive decline no longer significant (*p* > 0.05; Figure 2C). In total, 30 sets of variables were identified, of which 25 sets contained VGF, hippocampal sclerosis, and tangle measures (Figure 2C). Together with this core variable set, either *MIR132*, beta-amyloid, TAR DNA binding protein 43 (TDP-43), IGFBP5, or histone coacetylation modules could explain the mediation of AD-PRS to cognitive decline (Figure 2C). This implied that these variables were driven by different components of genetic risk factors for Alzheimer's dementia despite being associated with both AD-PRS and cognitive declines. To visualize how genetics aligned with certain traits, we projected their relationships onto 2-dimensional space using t-distributed stochastic neighbor embedding (t-SNE) (van der Maaten and Hinton, 2008), using their associations with the 457 SNPs in AD-PRS as a measure of distance. There were five major sets of traits, each of which were densely clustered together (Figure 2D). The trait group one (t1) was comprised of cognitive decline measures and clinical diagnosis of AD in both ROSMAP and IGAP, suggesting that these clinical signs of Alzheimer's dementia were affected similarly by 457 SNPs. Histone acetylation modules (t2), brain pathologies such as tangles and beta-amyloid (t3), VGF, syntaxin 1A (STX1A), and *MIR132* (t4), and TDP-43 and hippocampal sclerosis (t5) formed distinct clusters, respectively (Figure 2D), implying a differential influence of genetic risk factors on endophenotypes. This suggests that AD-PRS affects multiple molecular pathways that together lead to cognitive decline.

### Clustering risk variants finds underlying genetic architecture of Alzheimer's dementia phenotypes

The PRS concept treats genetic risks as a monolithic aggregate. While this perspective was sufficient to unite many weak variants and point at the molecular and neuropathologic features outlined above, there may be subgroups of genetic factors under the PRS aggregate. To investigate this possibility, we identified four SNP groups (Figure 2E and Supplementary Table 4) among the 457 SNPs in the AD-PRS by using SpeakEasy consensus clustering method. Each of these groups of SNPs exhibited a particular pattern of relationship to traits. The first group (s1) showed consistent effects across all traits. The second group (s2) did not demonstrate any associations with histone coacetylation. Conversely, the fourth group (s4) exhibited a specific effect on histone coacetylation. The SNPs in the third group (s3) had weak effects on all traits. These groups were independent of the significance levels of SNPs in IGAP study (Kruskal-Wallis test; *p* = 0.21). To investigate the distinctions among the molecular systems targeted by these four SNP groups, we conducted gene ontology (GO) enrichment analysis for genes located in *cis*-regions of the SNP groups using the binomial test (McLean et al., 2010). We identified 31 GO terms associated with one of the SNP groups (FDR < 0.05; Figure 2F and Supplementary Table 6). The GO terms related to immunity were enriched in s1, s2, and s3; ones associated with reactive oxygen species and lipid complex were enriched in s3; s4 was involved in phosphatidylinositol biosynthetic process. These biological processes could explain the distinct patterns of associations between the SNP groups and traits. Next, we examined whether incorporating the SNP groups into the PRS analysis could potentially improve the association of AD-PRS with mRNA modules and DNA methylation modules as previously shown in Figure 1. The AD-PRS was recalculated using the contents of each SNP group and jointly included in the regression model for all modules. Interestingly, the AD-PRS based on group s1 showed significant associations with modules -1 DNA methylation module and 5 mRNA modules using the significance threshold as shown in Figure 1 (Supplementary Figure 4). No relationships were found for other SNP groups. This further suggested that the SNPs in the s1 group have convergent effects on clinical phenotypes, neuropathologies, and multi-omic signatures in the ROSMAP cohorts.

### Polygenic risk increased histone acetylation in actively transcribed regions

**Figure 3:**
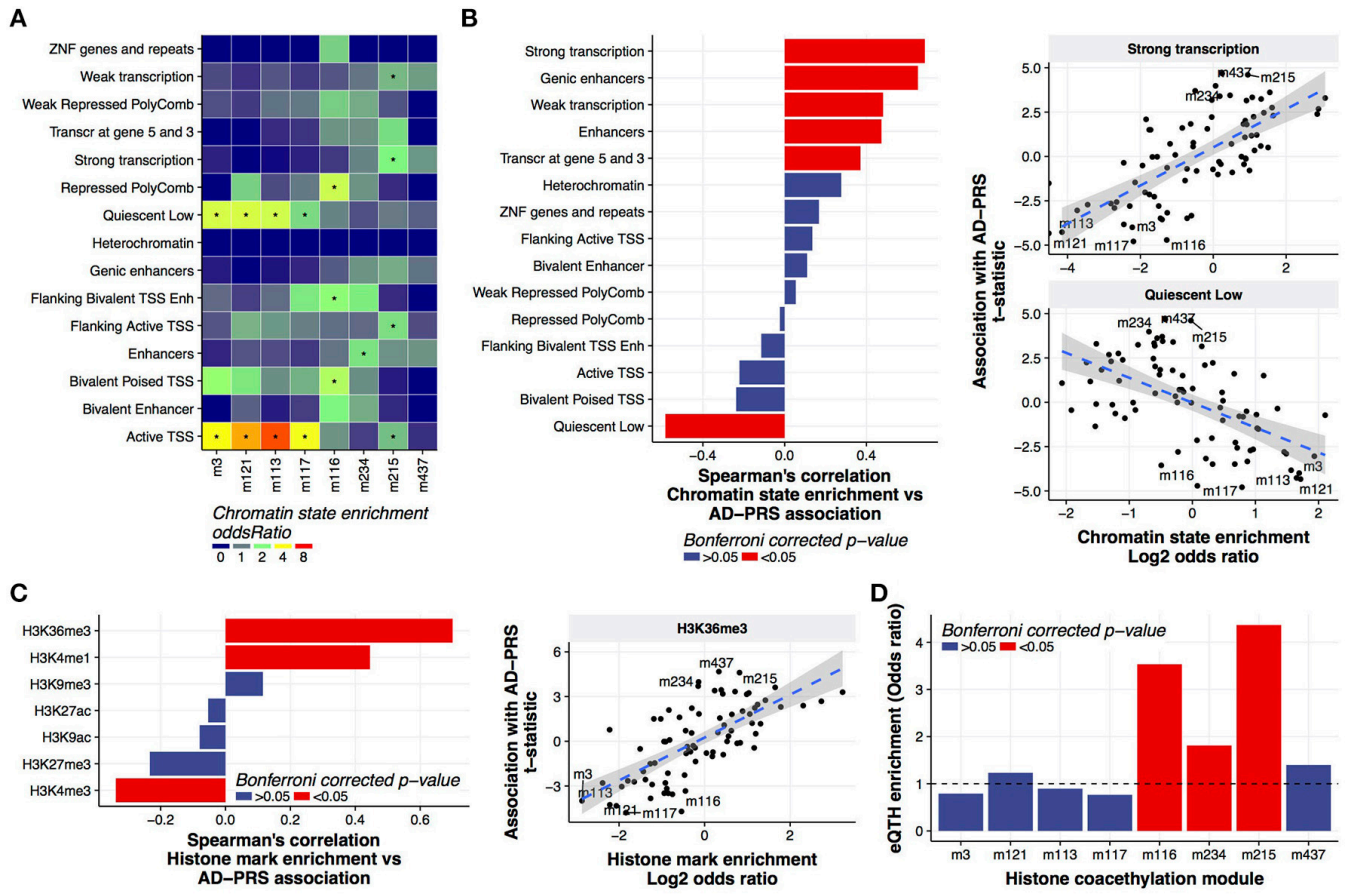
Characterization of histone coacetylation modules associated with AD-PRS **(A)**. Chromatin state enrichment of histone coacetylation modules. Overlap between histone coacetylation modules and 15 chromatin states from the mid-frontal gyrus was assessed using the Fisher's exact test via the LOLA method. An asterisk indicates Bonferroni-corrected *p* < 0.05 **(B)**. Correlation between chromatin state enrichment of coacetylation modules and their associations with AD-PRS **(C)**. Correlation between histone mark enrichment of coacetylation modules and their associations with AD-PRS. Overlap between histone coacetylation modules and seven histone marks from the mid-frontal gyrus was assessed via the LOLA method. The enrichment stats (log2 of odds ratio) were compared with association stats with AD-PRS **(D)**. Enrichment of eQTHs in modules. The enrichment was tested using the Fisher's exact test.

More than gene expression or DNA methylation, we found that histone acetylation was associated with the AD-PRS (Figure 1). To examine whether genetic risk variants for Alzheimer's dementia were enriched in coacetylated histone peaks, we conducted the LD score regression (Finucane et al., 2015) using the SNPs co-localized with the histone coacetylated modules or the *cis*- histone acetylation quantitative trait locus for the peaks in each module. No enrichment of Alzheimer's dementia heritability was observed in the PRS-associated modules (Supplementary Table 7) in both conditions, suggesting that the association with the AD-PRS occurs due to *trans*- rather than *cis*-regulatory mechanisms. To understand the genomic features involved in this regulation, we investigated the relationship between chromatin states and histone coacetylation modules. This revealed that the peaks of one set of modules - m3, m121, m113, and m117 - were significantly associated with quiescent (typically non-marked) chromatin and active TSSs, while the peaks of another set of modules - m215, m234, and m116 - were associated with strong transcription, enhancer, and Polycomb-repressed chromatin, respectively (Figure 3A). M437 was not significantly associated with any chromatin states, but its enrichment pattern was similar to m215. Based on this observation, we hypothesized that chromatin state could define the direction of the AD-PRS effect on acetylation. Indeed, the AD-PRS reduced the levels of acetylation in the modules located in quiescent chromatin regions and increased acetylation in the strongly transcribed and genic enhancer regions (Figure 3B). The chromatin states of strongly transcribed and genic enhancer regions are characterized by trimethylated histone H3 at lysine 36 (H3K36me3) (Bannister et al., 2005; Roadmap Epigenomics Consortium et al., 2015). As expected, the magnitude of colocalization of histone coacetylation modules with H3K36me determined the susceptibility to AD-PRS influence (Figure 3C). Despite coherent features of chromatin affected by the AD-PRS all pointing toward an effect on gene expression, we must in fact determine if AD-PRS-associated histone acetylation peaks actually drive gene expression in the brain. Therefore, we conducted a genome-wide correlation analysis between gene expression and histone acetylation (eQTH) within 1 Mbp of the TSS of those genes. We then evaluated the enrichment eQTHs in coacetylation modules. This revealed that histone coacetylation modules in active chromatin regions covaried significantly with the expression levels of nearby genes. Specifically we observed enrichment of eQTHs in modules m215 and m234 (Figure 3D); conversely, eQTHs tended to be depleted in modules located in quiescent regions such as m3, m113, and m117. Moreover, the genes that covaried with eQTHs in histone coacetylation modules that were up-regulated by the AD-PRS tended to show stronger associations with the AD-PRS than the genes correlated with eQTHs in modules down-regulated by AD-PRS (Wilcoxon test; *p* = 5.4e-07) or modules without associations with the AD-PRS (*p* = 3.0e-08) (Supplementary Figure 5). No significant GO terms were identified using genes associated with m215, m234, or m437, suggesting these modules do not carry out specific known biological functions. Overall, the AD-PRS elevated histone acetylation levels in the genome regions with high transcriptional activity, which might carry the AD-PRS effect to the transcriptome in aging brains.

## Discussion

Both early-onset and late-onset Alzheimer's dementia have a strong genetic basis (Gatz et al., 1997). While the genetic origin of early-onset Alzheimer's dementia is generally found in a small number of genes related to beta-amyloid protein processing; all of which are autosomal dominant at this time (Campion et al., 1995; Janssen et al., 2003), the genetic basis of the more common late-onset (sporadic) Alzheimer's dementia includes nearly 30 genes identified by GWAS and exome sequencing studies with genome-wide significance (Naj et al., 2011; Lambert et al., 2013; Cruchaga et al., 2014; Escott-Price et al., 2014; Jin et al., 2014; Vardarajan et al., 2015). Heritability for late-onset (sporadic) Alzheimer's dementia explained by genome-wide significant loci is limited (Ridge et al., 2013), and most of the genetic heritability for Alzheimer's dementia originates from multiple loci below the genome-wide significance threshold (Escott-Price et al., 2017). Given this genetic heterogeneity and the fact that few variants have a strong effect on risk, it may be challenging to obtain therapeutic effects in the broad population by targeting a single gene selected from susceptibility loci because of the collective weak contributions from multiple genes which cause Alzheimer's dementia. Therefore, the convergent effects from hundreds of independent risk variants identified in this study may be useful in identifying actionable disease mechanisms to target in drug treatments.

Our study identified a number of clinical, neuropathological and molecular features associated with the AD-PRS, some of which confirmed previous findings. Specifically, it was reported that the genetic risk for Alzheimer's dementia is correlated with the presence of neurofibrillary tangles and neuritic plaques in National Institute of Aging Alzheimer's disease centers (NIA ADC) study that includes our participants (Desikan et al., 2017). In addition, genetic risk was correlated with cognitive decline as reported in a study restricted to our participants (Felsky et al., 2018) and in the Alzheimer's disease neuroimaging initiative (ADNI) study (Mormino et al., 2016). Besides these replicated findings, we found novel associations with hippocampal sclerosis, TDP-43 pathology and expression levels of *MIR132*, protein abundance of VGF, STX1A, and IGFBP5, and histone acetylation in DLPFC. This extended our recent targeted analysis for APOE ε4 that showed the significant associations of APOE ε4 with hippocampal sclerosis and TDP-43 pathology (Yang et al., 2018) and revealed that non-APOE risk variants also contributed to the development of these pathologies. Furthermore, we explored potential molecular and neuropathologic factors mediating the relationship of the genetic risk for Alzheimer's dementia to cognitive decline. At least four factors, mainly hippocampal sclerosis, VGF, and tau-tangles, were necessary to fully explain the genetic effect on cognitive decline, suggesting AD-PRS has pleiotropic effects on multiple molecular pathways. Indeed, hippocampal sclerosis, VGF, and tau-tangles showed distinct patterns of SNP associations within the AD-PRS.

The AD-PRS-associated molecules play important roles in nervous systems, especially in the hippocampus. For example, *MIR132* reduces the brain expression level of a pathway involved in protein acetylation (Patrick et al., 2017), including histones, which may contribute to the effect of the PRS on the AD-associated histone acetylation patterns and the extensive chromatin remodeling that we have observed in Alzheimer's dementia (Klein et al., 2018). Further, the knockout of *MIR132* in mice impairs learning and memory (Hansen et al., 2016), and induces morphological changes in hippocampal neurons (Magill et al., 2010). VGF is a secreted neuropeptide that enhances memory formation and neurogenesis through potentiating brain-derived neurotrophic factor (BDNF) signaling in hippocampus (Thakker-Varia et al., 2014; Lin et al., 2015). The protein levels of VGF in cerebrospinal fluid are decreased in patients with AD compared to individuals with mild or no cognitive impairment (Duits et al., 2018). STX1A is a component of the presynaptic SNARE complex and located in the genome region that is responsible for Williams Syndrome characterized by intellectual or learning disability. The expression levels of STX1A in patient-derived lymphoblastoid cell lines explain 15.6% cognitive variation in patients with Williams Syndrome (Gao et al., 2010). In addition, the knockout mice of STX1A shows impairment of long-term potentiation in hippocampus (Mishima et al., 2012). IGFBP5 is an inhibitory binding protein for insulin-like growth factor 1 (IGF1) (Kalus et al., 1998). While the functions of IGFBP5 in cognitive phenotypes are not known, its target, IGF1, promotes hippocampal neurogenesis (Llorens-Martín et al., 2009). Thus, IGFBP5 itself also has a potential to regulate cognitive performance *in vivo*.

The AD-PRS increased the histone acetylation in the region where H3K36me3 modification is often observed in the reference epigenome panel (Roadmap Epigenomics Consortium et al., 2015). Histone modification of H3K36me3 ensures transcription of accurate forms of mRNAs by suppressing cryptic transcription through the reduction of histone acetylation (Strahl et al., 2002; Sen et al., 2015). Conversely, histone acetylation induces genome-wide cryptic transcription, which is epigenetically silenced in the normal state (Brocks et al., 2017). Thus, the gene expression correlated with histone acetylation might be the result of aberrant induction of cryptic transcription. Interestingly, the increase in cryptic transcription is associated with aging and short lifespan (Pu et al., 2015; Sen et al., 2015), and we have recently shown that splicing patterns are altered in reproducible ways in Alzheimer's dementia (Raj et al., 2017). Taken together, the increase in histone acetylation observed in the individuals with higher genetic risk for Alzheimer's dementia might impair transcriptional fidelity and advance the molecular age of brain.

Considering these complex relationships between Alzheimer's dementia genetics and endophenotypes, it is important to prioritize them from therapeutic perspective and understand the next steps. First, as there are multiple factors mediating AD-PRS effects on cognition, it may be necessary to develop multiple therapeutic agents to support healthy cognition. This points to the need to develop specific biomarkers, as brains of older adults have a tremendous range of neuropathologies with effects that vary greatly depending on coexisting pathologies (Boyle et al., 2018). However, placing older adults on multiple medications for the prevention of cognitive decline is not necessarily advisable without high probability of significant benefit that offsets risks and the cost of the medication. A PRS itself may be one component of future biomarkers, in conjunction with other types of data from non-invasive measurement technologies, which can be used to implement precision medicine solutions for individuals with Alzheimer's dementia (Hampel et al., 2017). Second, we need to elucidate the molecular and cellular mechanisms of how SNPs collectively affect endophenotypes. Specifically, although the subgroups of 457 risk SNPs for Alzheimer's dementia that had convergent effects on pathologies, protein, and histone modules were enriched with immune-related functions, the pathologies and molecular signatures themselves did not show the direct involvement of immune system. This suggests that there might be other proximal molecular or pathological consequences of the genetic risk for Alzheimer's dementia. Therefore, future experiments should be designed to fill this gap. We propose -omics profiling with finer spatial resolution, single-cell transcriptomics, and *in vitro* cell models assessing cellular phenotypes associated with the AD-PRS. The network models explaining the mediation of the AD-PRS will allow us to select the best targets for drug development.

The study has several limitations. First, as the sample size is not the same for all the data types, the strengths of the results are not directly comparable across all the variables. Related to this, we expect that increasing the sample size for -omics data would provide us with more power to detect molecules associated with the AD-PRS. This will be alleviated in the future as new -omics data are being generated from other participants. Second, in this study we focused on the DLPFC region. As molecules affected by the AD-PRS may differ across brain regions, examination of other brain regions may help to identify additional molecular signatures and molecular networks that are involved in the mediation of the AD-PRS. Such multi-region omics profiling is in process. Finally, ROS and MAP are volunteer cohorts with participants of mainly European descent and who are highly educated. Another study with a different ancestry composition and education levels would be beneficial to test the generalizability of these findings. Despite these shortcomings, availability of the multi-level data from a large number of individuals with multi-year cognitive evaluations allowed us to identify multiple factors that mediate the relationship between the AD-PRS to cognitive decline. Selected molecular and pathological features that we identified might be prime targets for interventions or biomarkers as they are common endpoints of the functionally diverse genetic loci associated with Alzheimer's dementia.

## Author contributions

ST contributed conception and design of the study. ST and CG performed the statistical analysis. ST, CG, SM, PD, and DB contributed to data generation and processing. ST, CG, SM, PD, and DB interpreted the result. ST and CG wrote the first draft of the manuscript. ST, CG, SM, PD, and DB contributed to manuscript revision, read and approved the submitted version.

### Conflict of interest statement

The authors declare that the research was conducted in the absence of any commercial or financial relationships that could be construed as a potential conflict of interest.
