# Supplementary material for: The Molecular and Neuropathological Consequences of Genetic Risk for Alzheimer's Dementia
Source: Front Neurosci. 2018 Oct 8;12:699. doi: 10.3389/fnins.2018.00699 (PMC6187226; doi:10.3389/fnins.2018.00699)
Supplement: Supplementary file 1 [file Table_1.pdf]

	Overall	DNAm	H3K9Ac	mRNA	miRNA	Protein
N	1272	651	631	494	655	765
Female (n, %)	847 (66.6)	408 (62.7)	414 (65.6)	307 (62.1)	417 (63.7)	502 (65.6)
Clinical dementia (n, %)	554 (43.6)	289 (44.4)	281 (44.5)	203 (41.1)	290 (44.3)	343 (44.8)
NCI/MCI (n, %)	690 (54.2)	350 (53.8)	339 (53.7)	282 (57.1)	353 (53.9)	411 (53.7)
AD (n, %)	471 (37.0)	233 (35.8)	226 (35.8)	168 (34.0)	236 (36.0)	283 (37.0)
Presence of gross infarctions (n, %)	458 (36.0)	232 (35.6)	225 (35.7)	174 (35.2)	239 (36.5)	277 (36.2)
Presence of microinfarcts (n, %)	377 (29.6)	175 (26.9)	170 (26.9)	127 (25.7)	178 (27.2)	216 (28.2)
Presence of Lewy bodies (n, %)	282 (22.2)	127 (19.5)	127 (20.1)	90 (18.2)	132 (20.2)	171 (22.4)
Age at death (mean (sd))	89.34 (6.56)	88.25 (6.63)	88.29 (6.51)	88.43 (6.63)	88.26 (6.53)	88.76 (6.42)
Years of education (mean, sd)	16.35 (3.60)	16.48 (3.52)	16.35 (3.52)	16.47 (3.47)	16.39 (3.56)	16.29 (3.51)
MMSE, last visit (mean, sd)	20.62 (9.40)	20.93 (9.14)	20.92 (9.19)	21.64 (8.84)	20.86 (9.33)	20.61 (9.33)
Global AD pathology (mean, sd)	0.74 (0.63)	0.70 (0.62)	0.71 (0.62)	0.66 (0.60)	0.71 (0.63)	0.73 (0.64)
Amyloid score (mean, sd)	4.16 (4.11)	3.49 (3.76)	3.61 (3.80)	3.54 (3.76)	3.56 (3.76)	4.42 (4.52)
Tangles score (mean, sd)	7.16 (8.67)	6.40 (8.08)	6.55 (8.24)	6.06 (7.67)	6.51 (8.15)	6.31 (7.54)

Supplementary Table 1. Cohort demographics.
