# Supplementary material for: The Molecular and Neuropathological Consequences of Genetic Risk for Alzheimer's Dementia
Source: Front Neurosci. 2018 Oct 8;12:699. doi: 10.3389/fnins.2018.00699 (PMC6187226; doi:10.3389/fnins.2018.00699)
Supplement: Supplementary file 8 [file Data_Sheet_1.pdf]

Title: The molecular and neuropathological consequences of genetic risk for Alzheimer's dementia

Authors: Shinya Tasaki[1,2], Chris Gaiteri[1,2], Sara Mostafavi[3], Philip L. De Jager[4,5], David A. Bennett[1,2]

Affiliations:

1 Rush Alzheimer's Disease Center, Rush University Medical Center, Chicago IL

2 Department of Neurological Sciences, Rush University Medical Center, Chicago, IL, USA

3 Department of Statistics, Department of Medical Genetics, University of British Columbia, Vancouver, BC, Canada

4 Center for Translational & Computational Neuroimmunology, Department of Neurology, Columbia University Medical Center, New York, NY, USA

5 Cell Circuits program, Broad Institute, Cambridge, Massachusetts, USA

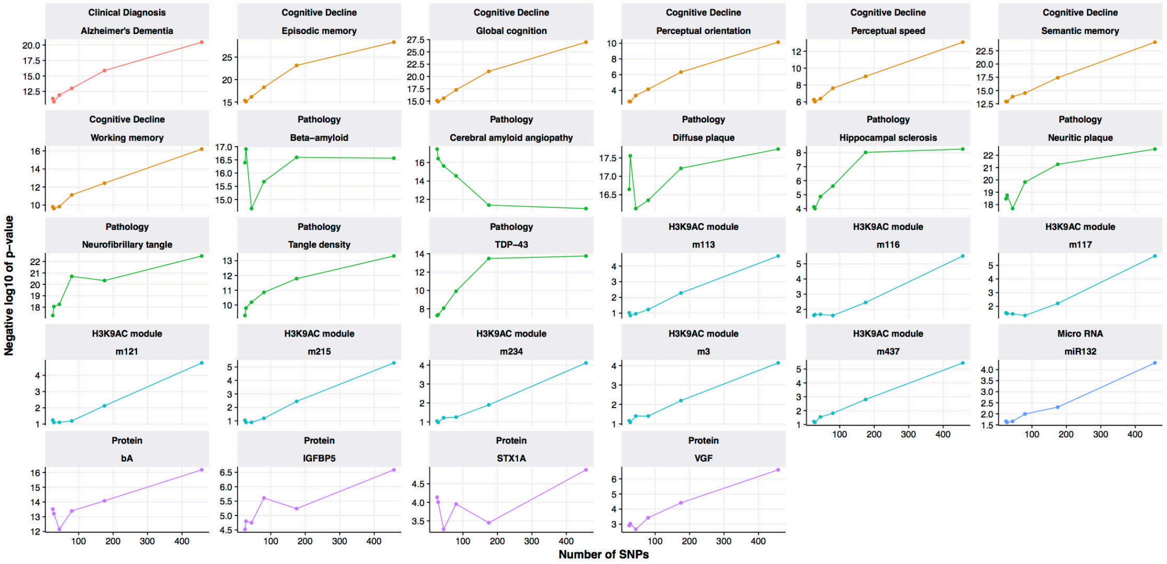

Supplementary Figure 1. Polygenic trend of AD-PRS-associated traits.

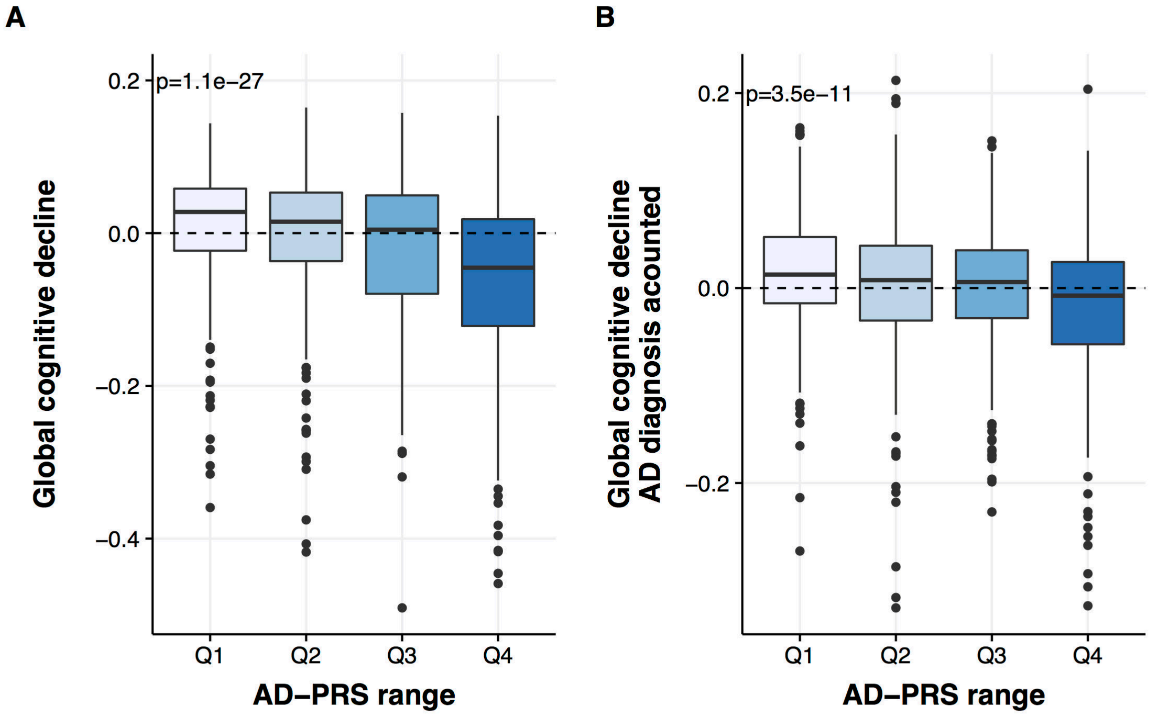

Supplementary Figure 2. Association of AD-PRS with global cognitive decline. (A) Global cognitive decline of individuals with different range of AD-PRS. ROS/MAP

participants were separated into four groups based on quantiles of AD-PRS. (B) Global cognitive decline of individuals with different range of AD-PRS with adjusting AD diagnosis.

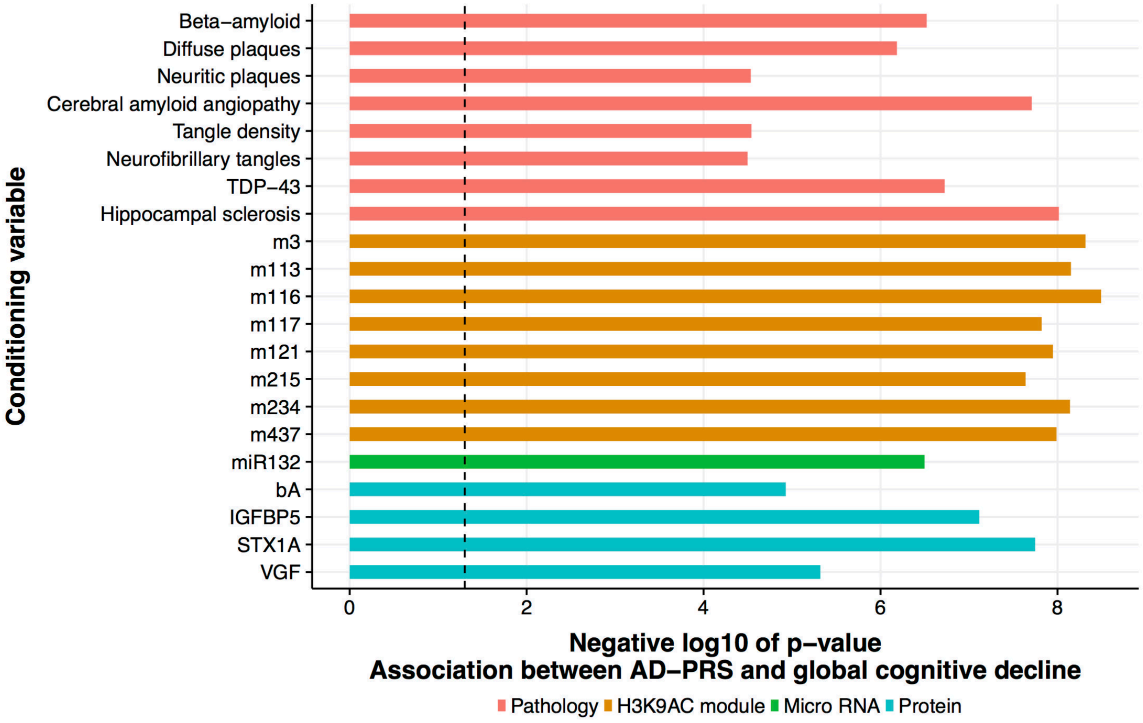

Supplementary Figure 3. Association between AD-PRS and global cognitive decline given an endophenotype. A bar plot represent p-values for association of AD-PRS with global cognitive decline given each endophenotype.

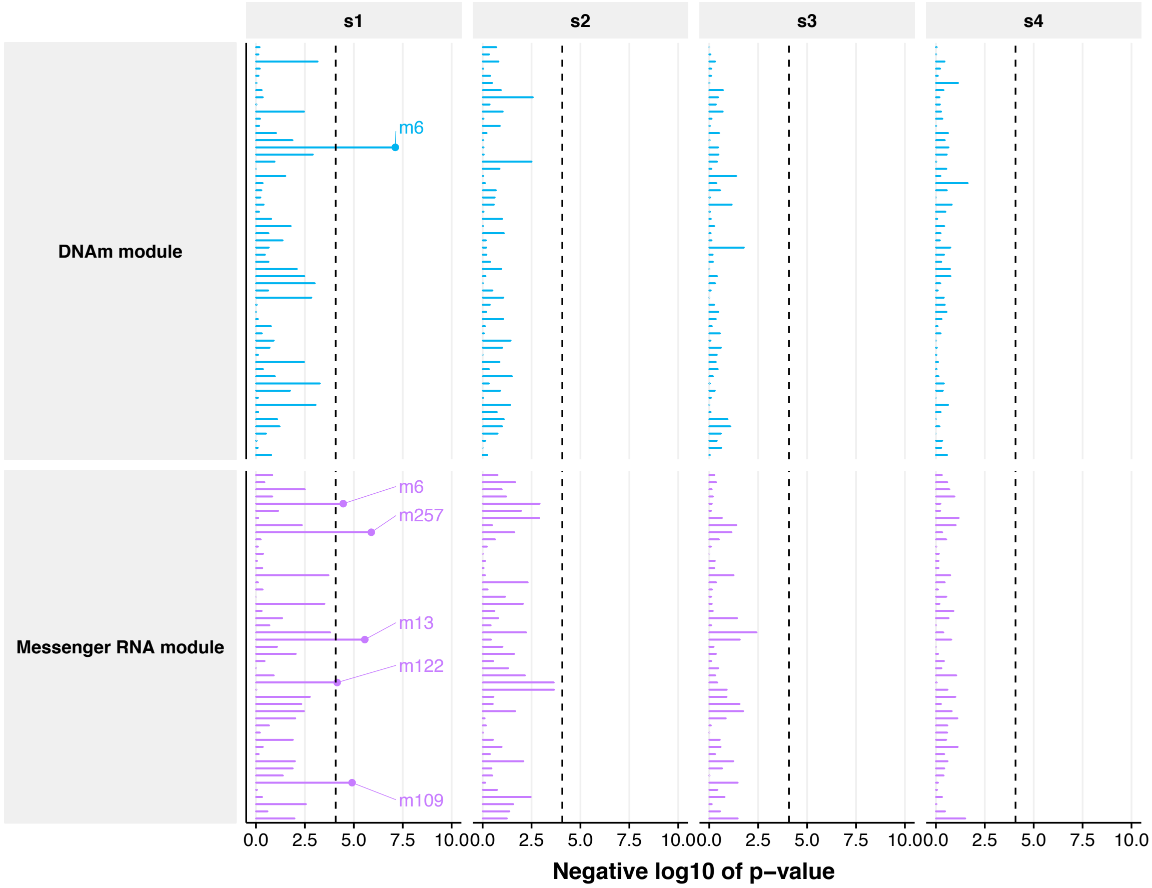

Supplementary Figure 4. Association of AD-PRS based on each SNP group with DNAm modules and mRNA modules. A dash line represents significance threshold that is identical to one in Figure 1.

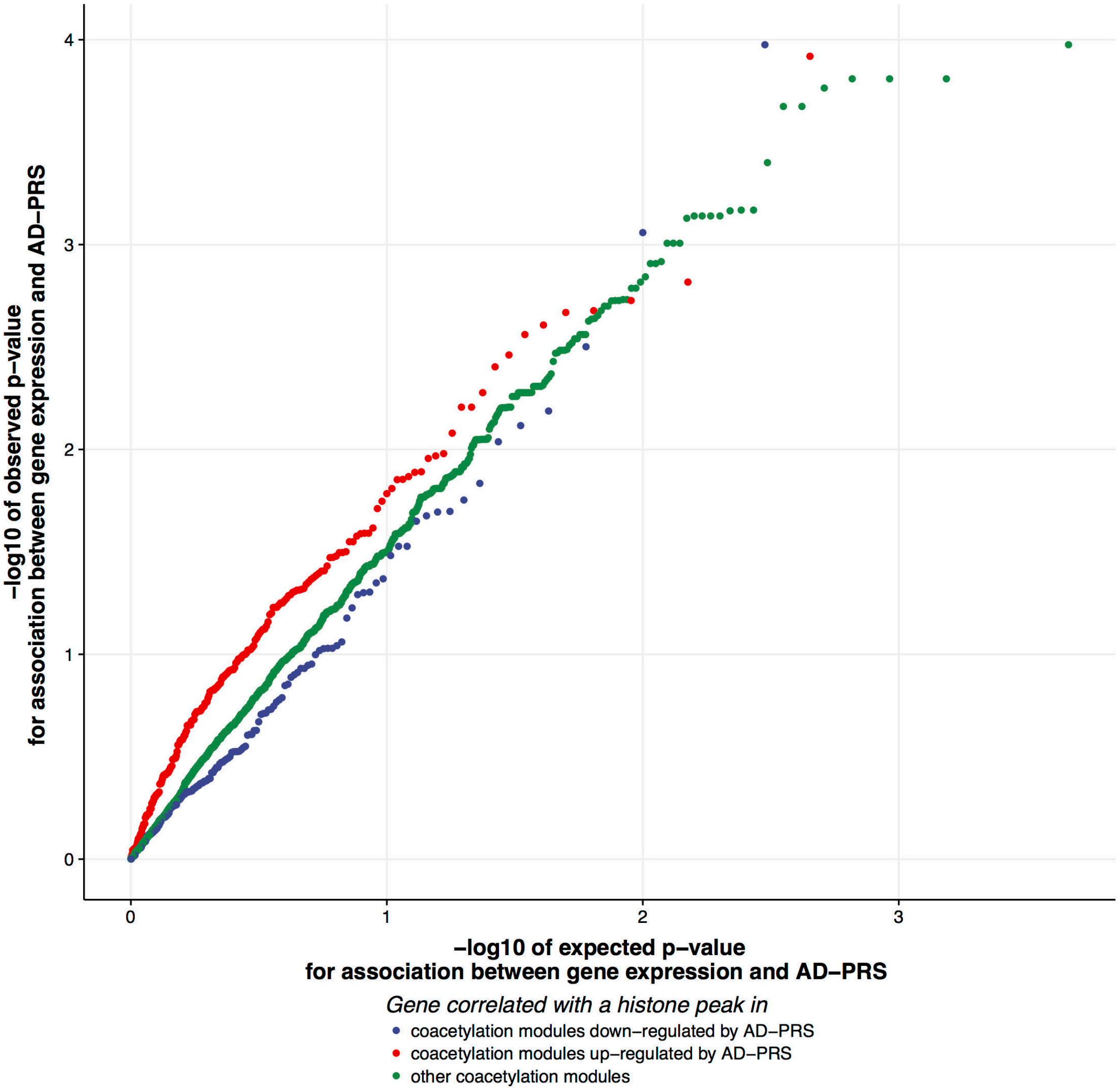

Supplementary Figure 5. Quantile-quantile plot for association of AD-PRS with gene expression grouped by associated eQTHs.
